# In vivo bio-distribution and acute toxicity evaluation of greenly synthesized ultra-small gold nanoparticles with different biological activities

**DOI:** 10.1038/s41598-022-10251-7

**Published:** 2022-04-15

**Authors:** Faizah S. Aljohani, Moaaz T. Hamed, Basant A. Bakr, Yahya H. Shahin, Marwa M. Abu-Serie, Ashraf K. Awaad, Hadir El-Kady, Bassma H. Elwakil

**Affiliations:** 1 Chemistry Department, Faculty of Science, Taibah University, Medina, Saudi Arabia; 2 Botany & Microbiology Department, Faculty of Science, Alexandria University, P.O. Box 21568, Alexandria, Egypt; 3 Zoology Department, Faculty of Science, Alexandria University, P.O. Box 21568, Alexandria, Egypt; 4 Medical Laboratory Technology Department, Faculty of Applied Health Sciences Technology, Pharos University in Alexandria, Alexandria, Egypt; 5 Medical Biotechnology Department, Genetic Engineering and Biotechnology Research Institute, City of Scientific Research and Technological Applications (SRTA-City), New Borg El Arab, Egypt; 6 Biochemistry and Molecular Biology Department, Center of Excellence for Research in Regenerative Medicine and Applications (CERRMA), Faculty of Medicine, Alexandria University, Alexandria, Egypt

**Keywords:** Antimicrobials, Microbiology, Diseases, Medical research, Drug development, Nanoscale materials

## Abstract

Ultra-small gold nanoparticles (Au-NPs) “≤ 10 nm diameters” have potent biomedical applications. Hence, the present study aimed to greenly synthesize ultra-small gold nanoparticles using Egyptian propolis extract. Different biological activities, in vivo bio-distribution and acute toxicity study were assessed. Results revealed that, Egyptian propolis extract can successfully synthesize the highly pure and stable ultra-small Au-NPs with average diameter 7.8 nm. In vitro antimicrobial and antimycobacterial activities revealed the powerful effect of the prepared Au-NPs. Moreover, the cytotoxic effect on human cancer cell lines revealed the potent inhibition of the cancer cells’ proliferation with high reactive oxygen species-mediated apoptosis induction. In vivo bio-distribution and acute toxicity studies were performed (10 and 100 mg/kg doses) in male albino rats. The ultra-small Au-NPs showed low or no toxicity upon using the Au-NPs low dose. The mean area accumulation (%) of the Au-NPs was higher in the liver, kidney, and brain tissues (4.41, 2.96, and 0.3 times, respectively) treated with high Au-NPs dosage compared to those treated with the low dose. Surprisingly, Au-NP accumulation in brain tissue was observed in the glial cells only. Accordingly, the low dose (10 mg/kg) of Au-NPs can be used safely in a variety of biomedical applications.

## Introduction

Gold nanoparticles (Au-NPs) are good choice for drug and gene delivery due to their wide variety of cargo^1^. Au-NPs can also be used as a delivery system in a phase I pharmacokinetic study for cancer drugs^2^. One of the major concerns in biomedical applications of Au-NPs is their tissue bioaccumulation which is the process by which an organism acquires a substance that has been deposited into its body^3^. However, Au-NPs safety and efficacy are still not clear^4^. The number of research papers that reported the Au-NPs bio-persistence and bioaccumulation are relatively low^5^. Toxicity assessment of Au-NPs after acute nanoparticles exposure has emerged as a global need especially after the divergent applications of Au-NPs as an anticancer, antibacterial, catalytic and drug carrier^6^. Serum analysis of enzymes and metabolites is a good indicator of hepatotoxicity and nephrotoxicity while the histopathological examination assesses the structural damage^7^ herein lies the importance of using both biochemical and histopathological analyses while assessing the acute Au-NPs toxicity.

Due to their wide variety of applications, various chemical, physical and biological methods have been used to produce Au-NPs^8^. Various biological synthesis methods have gained great attention due to their eco-friendly nature, simple methodology, cost effective and rapid synthesizing process^9^. Green Au-NPs synthesis approach can be achieved by variable reducing, capping agents and stabilizers^10^. For example: *Tamarindus indica*, *Benincasa hispida*, *Aloe vera*, *Rosa damascene*, *Cinnamomum camphora*, and seed extract of pomegranate^11–15^ have been used as reducing agents to greenly synthesize Au-NPs. Propolis (bee glue) is a resinous material collected by honeybee from the injured plant exudates and used as a constructing material of the bees’ hive. Several pharmacological activity reports have been concerned with propolis extracts for their hepatoprotective, antimicrobial, antioxidant, antitumor and anti-inflammatory activities^16^. Propolis composition differs according to the extraction season, climate and region^17^ however some propolis’ types have been used as gold reducing agents to form gold nanoparticles (Au^+3^ to Au^0^)^18–20^. Egyptian propolis is considered as distinct type with unique active molecules and unrevealed biological activities^21^. Hence, in the present work, Egyptian propolis was chosen as a novel gold reducing agent.

The aim of the present study was to greenly synthesize ultra-small gold nanoparticles using Egyptian propolis and exploring different biological activities of the prepared nanoparticles. The current work also aimed to evaluate the in vivo bio-distribution and acute toxicity of the greenly synthesized ultra-small gold nanoparticles in male Albino rats to prove their biocompatibility with biomedical applications.

## Material and methods

### Microorganisms

Different bacterial and yeast strains were under test namely *Acinetobacter baumannii, Bacillus cereus, Candida albicans*, *Klebsiella pneumoniae, Listeria monocytogenes*, Methicillin resistant *Staphylococcus aureus* (MRSA), *Proteus vulgaris*, *Staphylococcus aureus*, and *Staphylococcus epidermidis*. Five clinical isolates of *Mycobacterium tuberculosis* and one standard *M. tuberculosis* strain (ATCC 25177) were also under test. All the strains were kindly identified and provided by Surveillance Microbiology Department’s strain bank of the Main University Hospital, Alexandria.

### Chemicals & raw materials

The microbial culture media, and Gold chloride (Gold (III) chloride hydrate, 99.995% trace metals basis) were purchased from Sigma-Aldrich (MO, USA). In-vivo study reagents were purchased from BASF Co. (Ludwigshafen, Germany).

Propolis samples were collected during summer 2019 from Alexandria (31° 13′ N/29° 57′ E), Egypt. The samples were kept in sterile dark glass containers until further use.

### Propolis extraction and analysis

Propolis extraction was done by maceration in 99% ethanol (30% w/v). Continuous stirring in dark container was applied then each sample was sonified for 1 h at 72 °C^21^**.** The propolis ethanolic extract was filtered through Whatman filter paper No. 1 then dried using rotary evaporator. The propolis extract was analysed using GC–MS analysis^22^**.**

### Au-NPs synthesis

Two mM of gold chloride solution (1 mL) was added to 1 mL of propolis ethanolic extract (250 mg of the dried extract/1 mL dimethyl sulfoxide (DMSO))^20^. The mixture was stirred at room temperature for 2 h until the solution color turned to purple which indicated the Au-NPs formation. The mixture was left over-night at room temperature to complete the bio-reduction of Au-NPs using propolis extract^23^. The formed nanoparticles were centrifuged (20,000*g* for 20 min) then the precipitates were washed with deionized distilled water to eliminate any extract residues before further analyses.

### UV–visible spectroscopy

The Au/propolis mixture was assessed at different time intervals to confirm the nanoparticles formation through UV–Vis spectrophotometer at wavelength 450–850 nm^24^**.**

### Physicochemical characterization of the synthesized Au-NPs

Zeta potential, particle size (PS) and polydispersity index (PDI) of the synthesized nanoparticles were detected using Malvern Zetasizer following the dynamic light scattering (DLS) technique according to Elnaggar et al.^21^. FTIR (Perkin-Elmer R79521, USA) with 2 cm^-1^ resolution; wave number ranged between 4000 and 450 cm^-1^ during 64 scans was used to analyze the FTIR spectrum of the synthesized nanoparticles. On the other hand, the ultra-structure, shape and size of the synthesized Au-NPs were assessed through transmission electron microscope (TEM) (JEM-100 CX, JOEL, USA with resolution 3 nm at 30 kV)^24^. Au-NPs inflexibility, nature and shape were studied by scanning electron microscope (SEM) coupled with energy dispersive X-ray (EDX) (JEOL JSM 7610F equipped with X-Max^N^50 an Oxford Instruments X-ray detector)^25^.

Stability studies of Au-NPs in different physiological buffers and serum is important before biomedical applications, hence Au-NPs were incubated in PBS, 10% serum for 24 h, 48 h and 72 h and their stability and aggregation properties were assessed using zeta potential^26^**.**

### Antimicrobial activity of the synthesized Au- NPs

Different techniques were used to evaluate the antimicrobial activity of the synthesized Au-NPs. Firstly, bacterial suspensions of 1.5 × 10^6^ CFU/mL (0.5 McFarland) were prepared, then 100 μL of each bacterial suspension was swabbed over the surface of Müeller-Hinton agar (MHA) plate. Disc-diffusion method was carried out according to modified Kirby-Bauer technique to assess the antibacterial activity; each sterilized disc (Whatman No. 1/6 mm diameter) was saturated with 25 μL Au-NPs (20 mg/mL) then placed over the inoculated MHA plates. The incubation period lasted for 18 h at 37 ± 2 °C. Furthermore, the antibacterial activity evaluation was done by determining the minimum inhibitory concentration (MIC) and the microbial lethality curve to detect the optimum time and concentration needed for the tested nanoparticles to kill/inhibit the microbial vegetative cells^21^.

### Mycobacterium tuberculosis susceptibility testing

Proportion method on Löwenstein-Jensen medium was used according to European Centre for Disease Prevention and Control to estimate the proportion of the resistant tubercle bacilli to the prepared Au-NPs. Standard *M. tuberculosis* (ATCC 25177) and five *M. tuberculosis* clinical isolates were tested. Each diluted *M. tuberculosis* inoculum was inoculated on control media and Au-NPs containing media simultaneously. The tubercle bacilli growth on both media was compared then expressed as resistant percentage of the total population tested. McFarland standard No.1 was adjusted for each *M. tuberculosis* strain then 100 µL suspension was inoculated on both control and Au-NPs containing media. Results were recorded after 28 days (early reading) and 42 days (final reading) post-incubation. Colonies were counted on both types of media (the number of colonies on the Au-NPs containing media represents the proportion of the resistant bacilli in the inoculum). The percentage of resistant colonies was calculated according to the following equation:

$$Resistance\% = \frac{Number\;of\;colonies\;in\;drug\;containing\;slopes}{Number\;of\;colonies\;in\;drug\;free\;slopes} \times 100$$

The strain was considered resistant to Au-NPs if 1% of tuberculosis bacilli showed resistance to the Au-NPs incorporated media. If the resistant colonies to the drug appeared on the 28th day post-inoculation, then the result was reported as resistant, with no further reading needed. If the result on the 28th day proved sensitivity to the prepared Au-NPs, further incubation was done and the final reading was applied on the 42nd day for these sensitive strains^27^.

### Anticancer evaluation against colon, liver and breast cancer cells

#### Cytotoxicity assay using human cancer cell lines

Three human cancer cell lines (colon, liver and breast cell lines) were used to test the cytotoxicity of the ultra-small Au-NPs. The colon cancer cell line (Caco-2) was grown in DMEM (Lonza, USA) supplemented with 10% FBS, whereas the liver cancer cell line (HepG-2) and triple negative breast cancer cell line (MDA-MB 231) were grown in RPMI-1640 (Lonza, USAIn). All cancer cells (4 × 10^3^ cells/well) were transplanted into sterile 96-well plates. After 24 h, three cancer cell lines were treated with successive doses of the tested Au-NPs (40, 20, 10, 5, and 2.5 µg/mL) for 72 h at 37 °C in a 5% CO_2_ incubator. The viability of cancer cells was assessed after the incubation time^28^. After 24 h, each well was refilled with 20 µL of 5 mg/mL MTT (Sigma, USA) and the plates were incubated at 37 °C for 3 h. The MTT solution was then withdrawn, 100 µL DMSO was added, and the absorbance of each well was measured at 570 nm with a microplate reader (BMG LabTech, Germany) to assess the suppression of cancer cell growth after 72 h of incubation with Au-NPs. Using the Graphpad Instat software, the half maximum inhibitory concentration (IC50) values were determined. A phase contrast inverted microscope (Olympus, Japan) with a digital camera has also been used to analyze cellular morphological changes before and after treatment with the synthesized ultra-small Au- NPs.

#### Determination of cellular reactive oxygen species (ROS)

After 72 h incubation of serial concentrations of the synthesized Au-NPs with Caco-2, HepG-2 and MDA-MB 231 cells, the cellular ROS level was quantified by incubating with 5 μM of 2,7 dichlorofluorescin diacetate (DCFH-DA) for 30 min at 37 °C in the dark. After washing, the fluorescence intensity of the oxidized form of DCFH-DA was detected at excitation wavelength 488 nm and emission wavelength 530 nm, using fluorometer (BMG LabTech, Germany).

#### Quantitative determination of the relative change in the expression of proapoptotic (p53 and BAX) and anti-apoptotic (Bcl2) genes

Total RNAs were extracted then cDNA was synthesized from the untreated and Au-NPs (IC_50_)-treated Caco-2, HepG-2 and MDA-MB 231 cells using Gene JET RNA Purification and cDNA synthesis kits (Thermo Scientific, USA). Real time PCR was carried out using SYBR green master mix and specific primers (Forward/Reverse) were: 5′-ATGTTTTGCCAACTGGCCAAG-3′/5′-TGAGCAGCGCTCATGGTG-3′, 5′-CCGCCGTGGACACAGAC-3′/5′-CAGAAAACATGTCAGCTGCCA-3′ and 5′-TCCGATCAGGAAGGCTAGAGTT-3′/5′-TCGGTCTCCTAAAAGCAGGC**-**3′ for p53 and BAX and Bcl2 genes, respectively. The 2^−ΔΔCT^ equation was utilized to calculate the gene expressions changes.

### In vivo acute toxicity study

#### Rats and administrative dose

The purpose of this investigation was to assess the cytotoxic effect of the ultra-small Au-NPs in vivo. The current acute toxicity study was in accordance with ARRIVE guidelines and authorized by the Animal Care and Use Committee (ACUC), Faculty of Science, Alexandria University, in line with the International Principles for Laboratory and Care of the European Community Directive of 1986; AU/04/20/01/28/9/02. Thirty male albino rats (*Rattus norvegicus albinus*), 4 months old, with an average body weight of 390 ± 30 g, were housed (3 rats/cage) with a normal 12 h light/12 h dark cycle under standard temperature and humidity conditions for 30 days in an appropriately ventilated room with unrestricted access to food and water ad libitum^29^.The rats were divided into three groups according to the variation of nanoparticle doses to which they were subjected:

- **Group 1:** Rats were given 0.15 mL sodium chloride as a control (0.9%).
- **Group 2:** Rats were given 0.15 mL Au-NPs (10 mg/kg) body mass index (considered as low dose).
- **Group 3:** Rats were given 0.15 mL Au-NPs (100 mg/kg) body mass index (considered as high dose).

For two days, treated animals got intraperitoneal injections of the Au-NPs dosages every 24 h, whereas the control group received 0.9% sodium chloride doses.

#### Experimental protocol

The animals were given intraperitoneal injections of ketamine hydrochloride (100 mg/kg) and xylazine (10 mg/kg) to induce rats anaesthesia. Histopathological, blood biochemistry, and bioaccumulation investigations were performed 48 h after injection. For the next 30 days, each group's survival rate was observed and reported. The rats were anaesthetized by intraperitoneally administration of sodium pentobarbital (40 mg/kg) at the end of the experiment^29^.

#### Blood biochemical study

The cytotoxic impact of the synthesized Au-NPs was evaluated using a variety of biochemical assays. Blood was drawn from the retro orbital plexus of rats (n = 5). To obtain the serum sample, each blood sample was centrifuged for 20 min at 4000 g. An automated biochemical analyzer (Mindray BS-200, India) was used to determine liver functions (alanine aminotransferase (ALT), aspartate aminotransferase (AST), total bilirubin, albumin, alkaline phosphatase, and gamma-glutamyl transferase (GGT)), kidney functions (uric acid, creatinine, and urea), total cholesterol (high and low density lipoproteins), glucose, and total protein^30^**.**

#### Histopathological study

##### Light microscopic examination

After rats’ scarification, the liver, kidney, and brain were excised and weighed. Each tissue sample was washed with normal saline, fixed with 10% formalin, and then dehydrated with ethanol. Tissue samples were embedded in paraffin, sectioned into 5 m thick sections, deparaffinized, and stained with Ehrlich’s hematoxylin and eosin (H&E) stain^21^**.**

##### Imaging (localization) by confocal laser scanning microscopy

Deparaffinized tissue samples were mounted on commercially obtained charged slides, and dried at 60 °C in an oven for 1 h prior to staining. Tissues were stained with 10 μg/mL Hoechst 33342 (Abcam ab228551, Cambridge, UK) at room temperature in the dark for 5 min. The stained tissues were observed with the use of an inverted confocal microscope Leica DMi8 (Leica, Wetzlar, Germany) equipped using both 63× and 20× oil immersion objectives. The following acquisition wavelengths were used: Hoechst 33342, excitation 405 nm, emission 410–461 nm; red fluorescent Au-NPs, excitation 554 nm, emission 580–620 nm. At least five images of different tissues were randomly collected from each tissue. The images were captured and analyzed using ImageJ software. We used intensity profiles to exemplify fluorescent signal localization of Au-NPs with respect to nucleus. Briefly, intensity profiles were manually drawn with the Segmented Line tool for at least 10 cells for each tissue at different doses and analyzed with ImageJ software > Plot Profile^31^.

### Statistical analyses

Results were the mean of three trials and expressed as means ± standard deviation. One-way analysis of variance (ANOVA), Tukey’s test and unpaired T-tests (p < 0.05) were used to detect differences between the groups with the SPSS 13.0 statistical software. The means of the treatments were considered significant when 0.05 > p > 0.01.

## Results

### Egyptian propolis analysis

GC–MS analysis of Egyptian propolis extract revealed that heptacosane was the most abundant compound followed by Pregn-5-en-20-one, 11-(acetyloxy)-3,14-dihydroxy-12-(2-hydroxy-3-methyl-1-oxobutoxy) and Octacosanol (70.6, 34.9 and 34.8 respectively) (Table 1).

**Table 1:** GC–MS analysis of the Egyptian propolis sample.

Probable compound	Relative percentage (%)
Heptacosane	70.6
n-Hexadecanoic acid	15.3
trans-13-octadecenoic acid	13.4
R1-Barrigenol	18.6
Estra-1,3,5(10)-trien-17-one, 3-hydroxy-2-methoxy	14.6
4β-Methylandrostane2,3-diol-1,17-dione	27.2
Pregnan-20-one, 3,17-dihydroxy-, (3β,5β)	11.6
Octadecane, 3-ethyl-5-(2-ethylbutyl)	22.3
Pregn-5-en-20-one, 11-(acetyloxy)-3,14-dihydroxy-12-(2-hydroxy-3-methyl-1-oxobutoxy)	34.9
Cyclohexamine, *N*-n-butyl-1-(2-thionaphthenyl)	25.4
Octacosanol	34.8

### UV–visible spectroscopy analysis of the prepared Au-NPs

**Figure 1:**
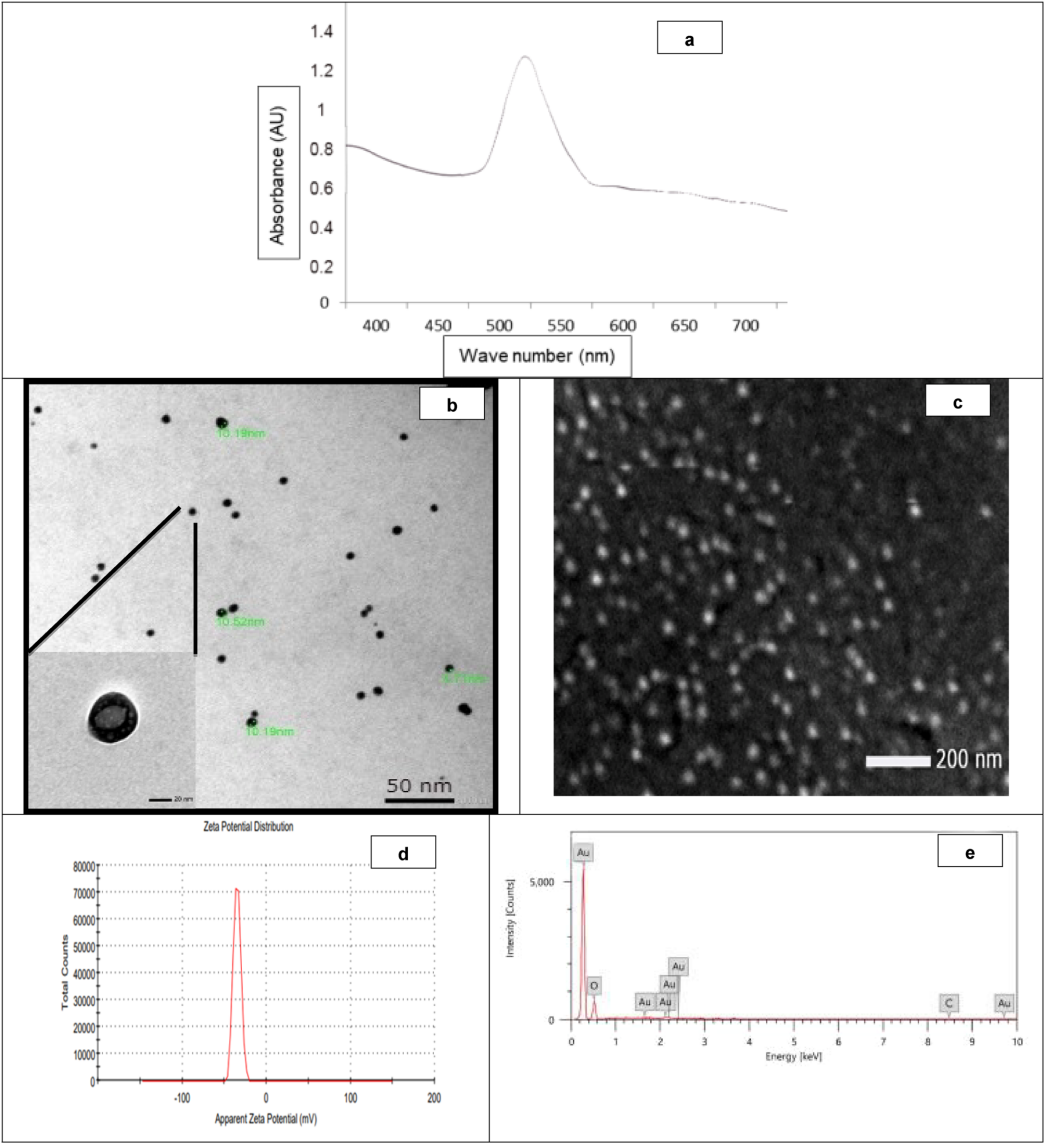

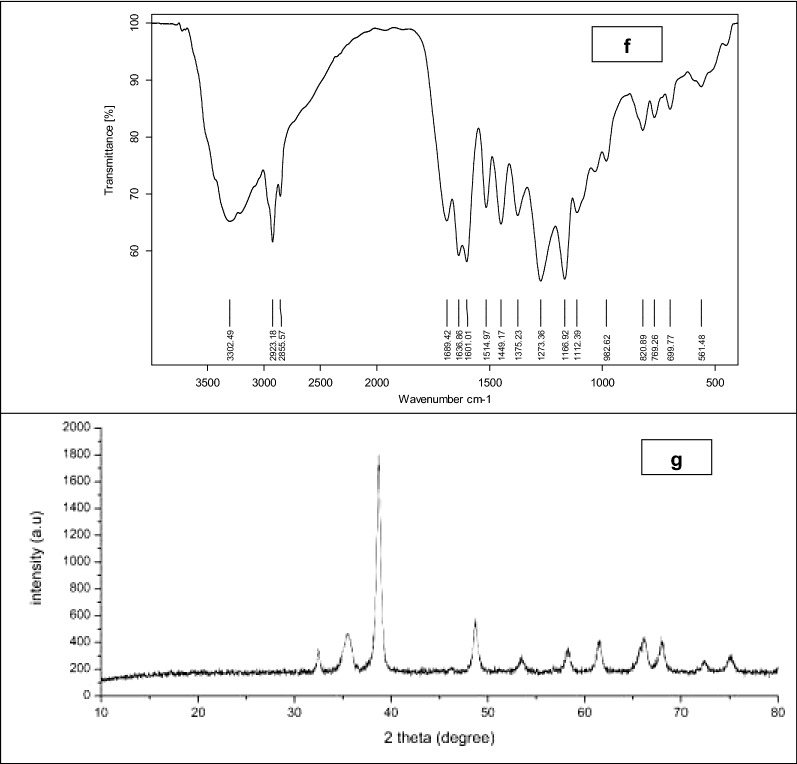
Au-NPs physicochemical characteristics; where (**a**) UV–Vis spectrum, (**b**) TEM analysis, (**c**) SEM analysis, (**d**) Zeta potential, (**e**) EDX, (**f**) FTIR analysis and (**g**) X-ray diffraction of the prepared nanoparticles.

The formation of nanoparticles was confirmed by the purple/pink color appearance after mixing the propolis extract and gold chloride solution (over-night). The colloidal solution of Au/propolis was scanned using UV–visible spectroscopy. Data in Fig. 1a revealed the characteristic peak of Au-NPs at 530–538 nm.

### Physicochemical characterization of the synthesized Au-NPs

#### Particle size, PDI, zeta potential, FTIR and XRD

The physical and chemical characteristics of the newly synthesized ultra-small Au-NPs revealed that the hydrodynamic diameter, PDI value and Z-potential were 15.4 nm, 0.385 and − 34.7 mV, respectively. The observed negative charge of Z-potential (Fig. 1d) could be attributed to the negative surface charge, due to the propolis anticipation as reducing with terminal carboxyl groups which was further confirmed through EDX and FTIR analyses. EDX analysis of the prepared Au-NPs was assessed and it was revealed that the Au atom% was 79.3 ± 0.23% while oxygen and carbon atoms% were 20.64 ± 0.31 and 0.06 ± 0.01% respectively which proved the nanoparticles purity (Fig. 1e).

FTIR analysis may prove the aforementioned theory by providing information about the functionalized groups that maybe attributed in the Au-NPs reduction process. A strong band at 3330–3270 cm^-1^ was observed which represents a terminal alkyne with C–H stretch and it was confirmed by the C–H bending vibration at 700–610 cm^-1^ (Fig. 1f). The presence of CH_2_ stretching vibrations bands in the propolis extract at 2915 and 2850 cm^-1^ which were shifted in the formed Au-NPs to 2923 and 2855 cm^-1^ respectively which was also observed in EDX analysis. This may indicate the effect of some chemisorbed functional groups to prevent the nanoparticles aggregation (Fig. 1f and Fig. S1).

On the other hand, XRD reveals three characteristics peaks for Au-NPs at 2θ = 35^0^, 38^0^, and 48^0^ respectively corresponding to 111, 200, and 220. The high intense peak (111) and (200) relative to (220) which was observed in the powdered sample proved the spherical shape of the synthesized nanoparticles. The XRD data and HR-TEM results confirmed that samples were pure crystalline and without impurities (Fig. 1g). Nanoparticles characteristics (IR and XRD analysis) were compared to the propolis extract alone (Fig. S1 and Fig. S2) to assess the gold nanoparticles’ successful formation.

#### Transmission (TEM) and scanning (SEM) electron microscopic examination

The ultra-structure, size and shape of the prepared Au-NPs were examined by transmission and scanning electron microscope. It was found that the gold nanoparticles have a perfect spherical shape with average ultra-small size of 7.8 ± 2 nm (Fig. 1b,c).

#### Au-NPs stability

The stability of the synthesized Au-NPs was evaluated by monitoring the zeta potential changes in serum, phosphate buffer solution at pH 6, 7.4 and 8. The present results (Table 2) proved that ultra-small Au-NPs were highly stable in biological fluids at physiological pH and in serum. During the different incubation periods, the zeta potential measurements slightly increased with time.

**Table 2:** Zeta potential (mV) of Au-NPs in PBS, 10% serum for 24 h, 48 h and 72 h.

	24 h	48 h	72 h
PBS pH 6	− 22.5 ± 2.7	− 23.0 ± 3.4	− 23.7 ± 1.6
PBS pH 7	− 46.4 ± 1.1	− 46.9 ± 0.7	− 49.1 ± 4
PBS pH 8	− 29.2 ±	− 29.0 ± 2.5	− 29.6 ± 0.9
Serum	− 47.3 ± 0.5	− 48.7 ± 2.8	− 48.9 ± 1.3

### Antimicrobial activity of the synthesized Au-NPs

**Figure 2:**
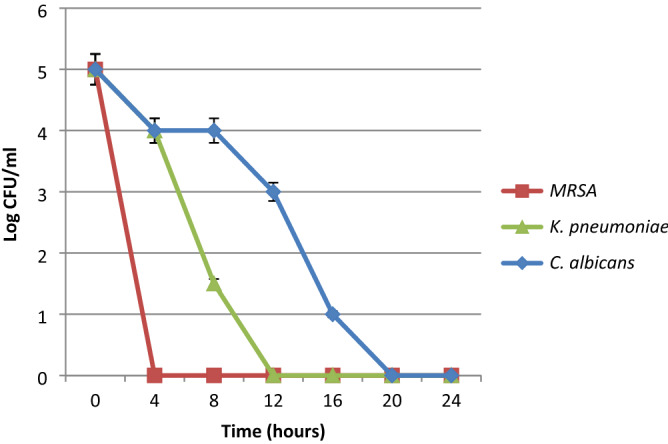
Time lethality curve of the ultra-small Au-NPs against *Candida albicans,* MRSA and *K. pneumonia*.

Results revealed that, the prepared Au-NPs had a significant antimicrobial activity with average inhibition zone diameter 22.7 mm (Table 3). The observed antimicrobial activity could be attributed to the nanoparticles small size and great stability. However, the prepared nanoparticles showed significantly high antibacterial activity against Gram-positive strains specially *Staphylococcus epidermidis* (4 µg/mL MIC value and 45 mm inhibition zone diameter). MRSA, *K. pneumoniae* and *C. albicans* were the most resistant strains hence they were chosen for further analysis. Microbial lethality curve revealed that the ultra-small Au-NPs had a significantly effective microbial eradication activity. Results in Fig. 2 demonstrated that the complete eradication of MRSA cells occurred after 4 h incubation with Au-NPs compared to *C. albicans* and *K. pneumonia* which needed 20 and 12 h respectively to be completely inhibited.

**Table 3:** The antimicrobial activity of the synthesized Au-NPs.

Tested microorganism	Inhibition zone diameter (mm)	MIC (µg/mL)
*Candida albicans*	17	64
*Bacillus cereus*	25	16
*S. aureus*	30	16
*S. epidermidis*	45	4
MRSA	20	64
*Acinetobacter baumannii*	20	32
*K. pneumonia*	13	128
*Listeria monocytogenes*	20	32
*P. vulgaris*	15	128

### Mycobacterium tuberculosis susceptibility testing

*Mycobacterium tuberculosis* (TB) remains a very contagious challenging microbe to control around the world. Although several biomedical applications have been reported for Au-NPs, the researches reporting the anti-TB activity of ultra-small gold nanoparticles are very scarce. Herein, different *M. tuberculosis* clinical isolates and one standard strain of *M. tuberculosis* (ATCC 25177) were tested for their susceptibility to the prepared Au-NPs. It was revealed that all the tested clinical isolates showed more than 1% growth on Au-NPs (1 mg/mL) containing media when compared to the control media indicating that the tested *M. tuberculosis* clinical strains were resistant. However by increasing the Au-NPs concentration (10 mg/mL), the inhibitory effect increased to reach 99–90% growth inhibition of the tubercle bacilli population when compared to the control tubes (Table 4). On the other hand, the ultra-small Au-NPs effectively inhibited the growth of the standard strain *M. tuberculosis* (ATCC 25177) (0.6% resistance).

**Table 4:** The TB resistance percentage against the prepared ultra-small Au-NPs.

Tested strains	Resistance (%)	
	Au-NPs (1 mg/ml)	Au-NPs (10 mg/ml)
*M. tuberculosis* ATCC 25177	59	0.6
*M. tuberculosis* 1	64	1
*M. tuberculosis* 2	73	5
*M. tuberculosis* 3	70	6
*M. tuberculosis* 4	77	10
*M. tuberculosis* 5	72	2

### Anticancer efficacy of Au-NPs

#### Growth inhibition potency on human cancer cell lines

**Figure 3:**
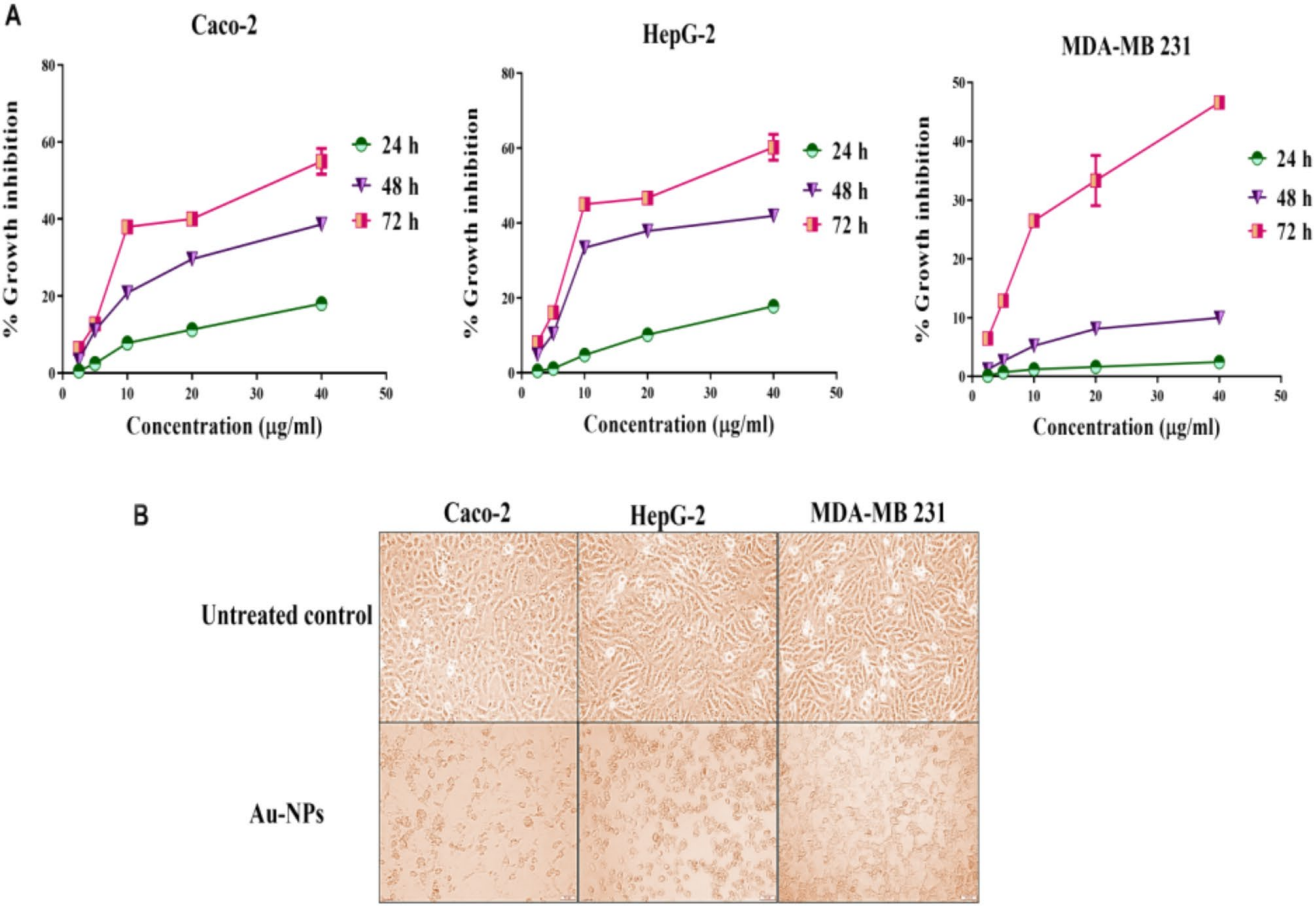
Cytotoxicity of the ultra-small Au-NPs on human cancer cells against human cancer cell lines (Caco-2, HepG-2 and MDA-MB 231). (**A**) The percentage of growth inhibition of each cancer cell line after 24 h, 48 h, and 72 h incubation. (**B**) Morphological alterations of Au NPs-treated cancer cells in comparison to the untreated control cells (Magnification × 200). All data are expressed as mean ± SEM.

Results in Fig. 3A,B indicated that the ultra-small Au-NPs exhibited potent growth inhibitory effect against the examined human cancer cell lines. During 72 h of incubation with serial dilutions of Au-NPs, the percentages of growth inhibition of each human cancer cell line were estimated. It was found that Au-NPs inhibited the growth of all the tested cancer cell lines in dose-and time-dependent manner as shown in Fig. 3A. Accordingly, the maximum inhibition of cancer cell proliferation was recorded at 72 h (Fig. 3A). Au-NPs exhibited potent cytotoxicity for inhibiting the proliferation of Caco-2, HepG-2 and MDA-MB 231 cells with low IC50 (16.55 ± 1.55, 10.92 ± 1.13, 23.84 ± 2.98 µg/mL, respectively). The morphological collapse of Au-NPs-treated human cancer cells supported their powerful anticancer activity (Fig. 3B).

#### Elevation of cellular ROS in Au NPs-treated cancer cells

**Figure 4:**
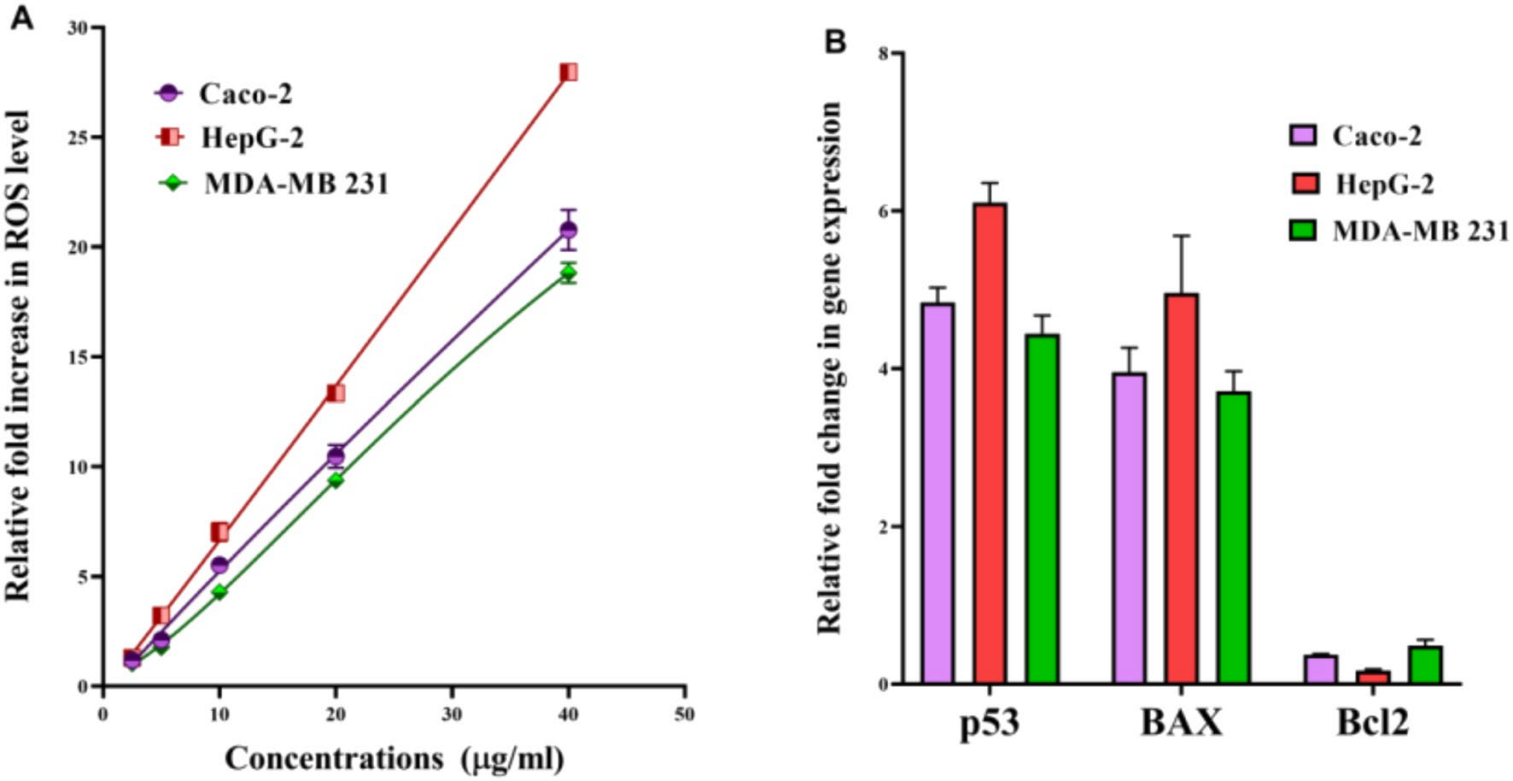
Prooxidant effect-mediated apoptotic activity of Au NPs human cancer cell lines (Caco-2, HepG-2 and MDA-MB 231). (**A**) The relative fold increase in ROS level in human cancer cell line after 72 h incubation with serial concentrations of Au NPs. (**B**) Relative change in p53, BAX and Bcl2 expression in IC_50_ of Au NPs-treated cancer cells. All data are expressed as mean ± SEM.

The change in redox status of the treated human cancer cells was detected based on the level of the oxidized fluorescence DCF. Figure 4A illustrates that Au-NPs caused a dose-dependent elevation in ROS in all studied cancer cell lines. At 20 µg/mL of Au-NPs, ROS level was increased by 10.99 ± 9.96 13.34 ± 0.09 and 9.37 ± 0.178 folds in Caco-2, HepG2 and MDA-MB 231 cells, respectively. The intracellular ROS content of the treated HepG2 with 10 µg/mL Au NPs was elevated by 7.03 ± 0.402 folds.

#### Stimulation of apoptotic genes with suppression of oncogene

The potential of Au-NPs for inducing apoptosis was assessed using QPCR which measured the relative change in expression of key proapoptotic and antiapoptotic genes. Figure 4B demonstrates the proapoptotic potency of Au NPs via enhancement of the expression of p53 by 4.84, 6.10 and 4.44 folds in the treated Caco-2, HepG2 and MDA-MB 231 cells, respectively. Subsequently, BAX expression was increased by 3.95, 4.96 and 3.71 folds, respectively. Meanwhile antiapoptotic gene (Bcl2) expression was down regulated by 2.67, 5.89 and 2.05 folds, respectively (Fig. 4B).

### In vivo acute toxicity study

#### Biochemical study

The results of the biochemical study of different rat groups receiving normal saline (Group 1), 10 mg/Kg Au-NPs (Groups 2) and 100 mg/kg Au- NPs (Groups 3) were presented in Table 5. It was revealed that Group 2 had minimum signs of hepato- and nephrotoxicity while Group 3 showed a significantly higher level of hepatotoxicity accompanied by a loss of appetite. The mentioned results proved that the Au-NPs toxicity is dose-dependent. However, no mortality was observed during the experimental period, creatinine and total cholesterol were significantly affected upon Au-NPs administration whether in low or high dose.

**Table 5:** Biochemical tests in serum samples of different rats groups.

Dose/test	Group 1 (control group)	Group 2 (received 10 mg/kg)	Group 3 (received 100 mg/kg)
Urea (mg/dl)	6.63 ± 1.47	17.30 ± 0.956	18.40 ± 0.17
Creatinine (mg/dl)	0.70 ± 0.63	1.6 ± 0.47	1.8 ± 0.94
Glucose (mg/dl)	80.10 ± 0.11	66.9 ± 0.09	101.1 ± 0.04
Cholesterol (md/dl)	19.6 ± 0.02^a^	50.70 ± 0.03^a^	130.0 ± 0.06^a^
Total protein (mg/dl)	3.5 ± 0.41	4.2 ± 0.08	4.9 ± 0.23
Albumin (g/dl)	3.90 ± 0.90	2.0 ± 1.73	2.9 ± 1.46
Uric acid (g/dl)	0.6 ± 0.37^b^	1.4 ± 0.51^b^	1.8 ± 0.48^b^
Total Serum Bilirubin (mg/dl)	0.20 ± 0.01	0.5 ± 0.01	0.7 ± 0.03
Alk Phos (U/L)	54.00 ± 1.49^C^	67.2 ± 1.73^C^	90.9 ± 1.97^C^
GGT (U/L)	12.3 ± 2.80^d^	23.5 ± 1.32^d^	70.00 ± 0.77^d^
ALT (U/L)	11.00 ± 0.21^e^	15.1 ± 0.13^e^	69.2 ± 0.61^e^
AST (U/L)	27.00 ± 0.92^f^	168.1 ± 0.45^f^	705.2 ± 0.28^f^

All values are expressed as mean ± SD. Different letters of the same column are significantly different at p < 0.05.

#### Toxicity, biodistribution and localization study of Au-NPs

##### Histopathological study

**Figure 5:**
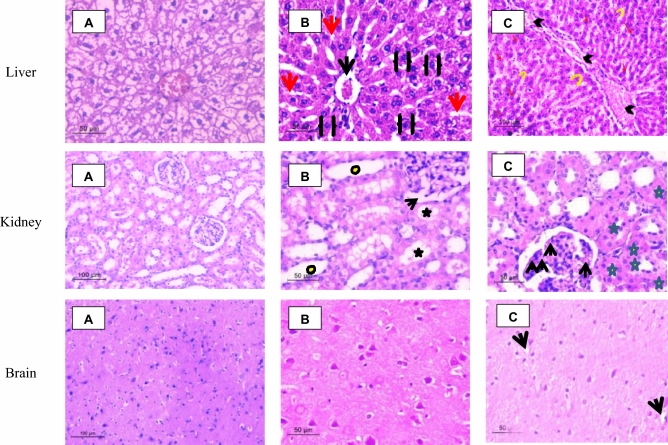
Light micrographs in liver tissue where (**A**) Liver sector of control rat showed normal tightly packed cords of hepatocytes with rounded vesicular nuclei, (**B**) Liver sector of low dose treated group (Group 2) showed hepatocytes radiating from central vein with normal endothelial cells. Dilated congested central vein (black arrow), Dilated blood sinusoids (red arrows) and Ballooned hepatocytes (black brackets) (**C**) Liver sector from high dose treated animals illustrated indefinite cell boundaries which confirm hepatocytes damage; severe congestion in central vein (black head arrow), nuclear changes (red arrows) and blood sinusoids congestion (yellow bent arrows). While, in kidney where (**A**) Light renal micrographs illustrate control rat received normal saline demonstrating normal histological architecture. (**B**) Light renal micrographs of low doses treated rats (Group 2) demonstrating most of convoluted tubules were apparently less or more normal (yellow and black Strikes) and normal architecture of bowman's capsule with space lining (arrow). (**C**) Light Renal micrographs of high dose treated rats (Group 3) reveling normal architecture of bowman’s capsules with little glomerular congestion or inflamed glomeruli (arrows), normal renal tubules with few number of necrotic ones (yellow star). Lastly, brain cerebral cortex illustrate (**A**) control rat (received normal saline) (**B**) Au-NPs low dose treated (Group 2) demonstrating normal histological architecture. (**C**) Au-NPs high dose treated (Group 3) demonstrating only two narrow vacuoles (black arrow).

*Liver* Histological examination of the control group showed normal structure of the liver, each hepatic lobule has a central vein with hepatic cords arranged in radiating shape embedding blood sinusoids which appeared as narrow spaces lined with flattened endothelial cells. Normal hepatocytes were observed polyhedral in shape with centrally located nuclei (Fig. 5A). On the other hand; liver sector of low dose treated group (Group 2) showed dilation of the central vein accompanied with severe hepatic sinusoids congestion. Some of hepatocytes were ballooned with vacuolated cytoplasm. Histopathological examination of the liver sector of high dose treated animals (Group 3) showed severe tissue toxicity mainly presenting as severe congestion in the central vein surrounded by mononuclear cellular infiltration. Many nuclear changes were detected including nuclear division, nuclear eccentricity, pyknosis, necrosis, and karyorrhexis. Moreover, mild multifocal infiltrations of inflammatory cells between the areas of hemorrhages were detected accompanied with indistinct cell boundaries (Fig. 5B,C).

*Kidney* Normal architecture of renal tubules and Bowman’s capsules was observed in the Kidney of control group (Group 1); in which Normal spherical nuclei were observed in cuboidal cells of proximal convoluted tubules with narrow lumina. Distal convoluted tubules had wide lumina with central or apical spherical nuclei (Fig. 5A). On the other hand, histological examination of kidney tissues in low dose treated group (Group 2) revealed no significant alteration of the normal histological structures (in the control group) except the presence of mild degeneration in the lining epithelium of the proximal convoluted tubules. Compared to previous results; the renal tissues of high dose treated rats (Group 3) also did not exhibit any signs of toxicity either hemorrhages or severe tissue damages but mild congestion of the glomerular capillaries was shown with few necrotic renal tubules (Fig. 5B,C).

*Brain* Histological changes in the cerebrum of experimental rats examined under light microscopy revealed no obvious damage or inflammation in brain cerebral cortex. Cerebral cortex of all experimental rats revealed normal cyto-architecture of neurons probably reflecting that Au-NPs did not cross the blood brain barrier (BBB) (Fig. 5A–C) as confirmed by confocal microscopy results.

##### In vivo biodistribution and localization of gold NPs by confocal microscopy

To evaluate the ultra-small Au-NPs biodistribution, confocal fluorescence microscopy was used to examine the nanoparticles (NPs) fluorescence in the main organs (liver, kidney, and brain), which was found to be dose- and tissue type-dependent. The cell layer was monitored through the nuclear staining (blue), while the NPs were monitored through scattering signal (red). By evaluating the 3D reconstruction cross section of the cell monolayer, a more complete picture of the NPs dosing was obtained. The Au-NPs showed adherence to the bottom of the cover slip (due to sedimentation of the NPs over time) as well as dispersion throughout the cell layer, indicating the NP incorporation.

**Figure 6:**
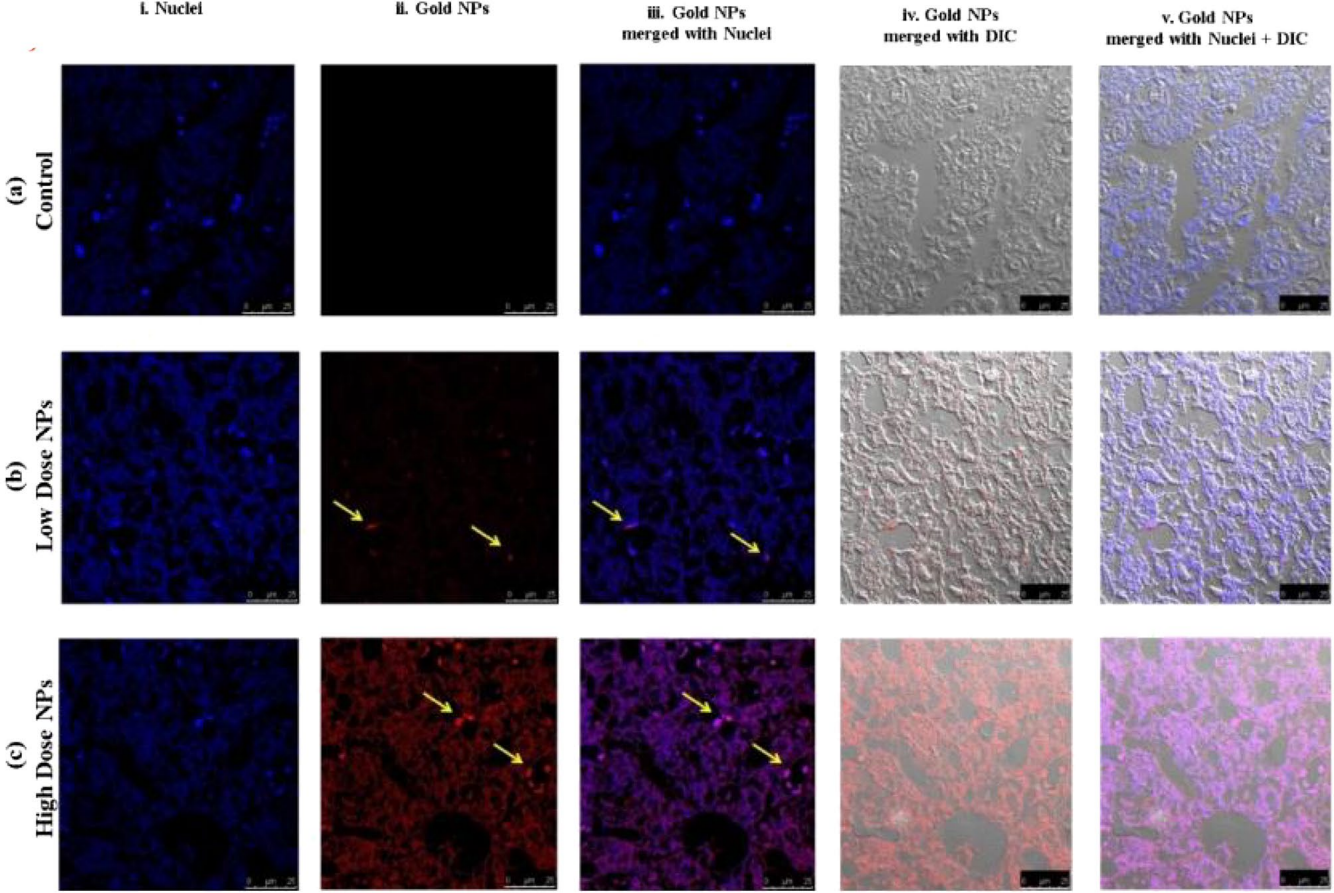
In vivo biodistribution and localization of Au-NPs at different doses (High & Low) in rat liver tissues. The Au-NPs were clearly visible in the cytoplasm and nucleus of liver tissue cells, especially in the sinusoidal macrophages (Kupffer cells) indicating that the macrophages were central in the elimination process of Au-NPs from the body (yellow arrows). (i) Blue: Hoechst 33342-stained nuclei. (ii) Red: Gold NPs. (iii) Merged Image from Gold NPs and Nuclei. (iv) DIC mode merged with Gold NPs (v) DIC mode merged with Gold NPs and nuclei. λ_max_ = 505 nm (Hoechst 33342), 555 nm (red autofluorescence from gold NPs), 473 nm (Differential interference contrast [DIC]). (Confocal Laser Scanning Microscope × 63, Scale bar: 25 μm, Scan Mode = XYZ Unidirectional X, Scan Speed = 400 Hz, Pinhole Diameter = 137.1 μm).

**Figure 7:**
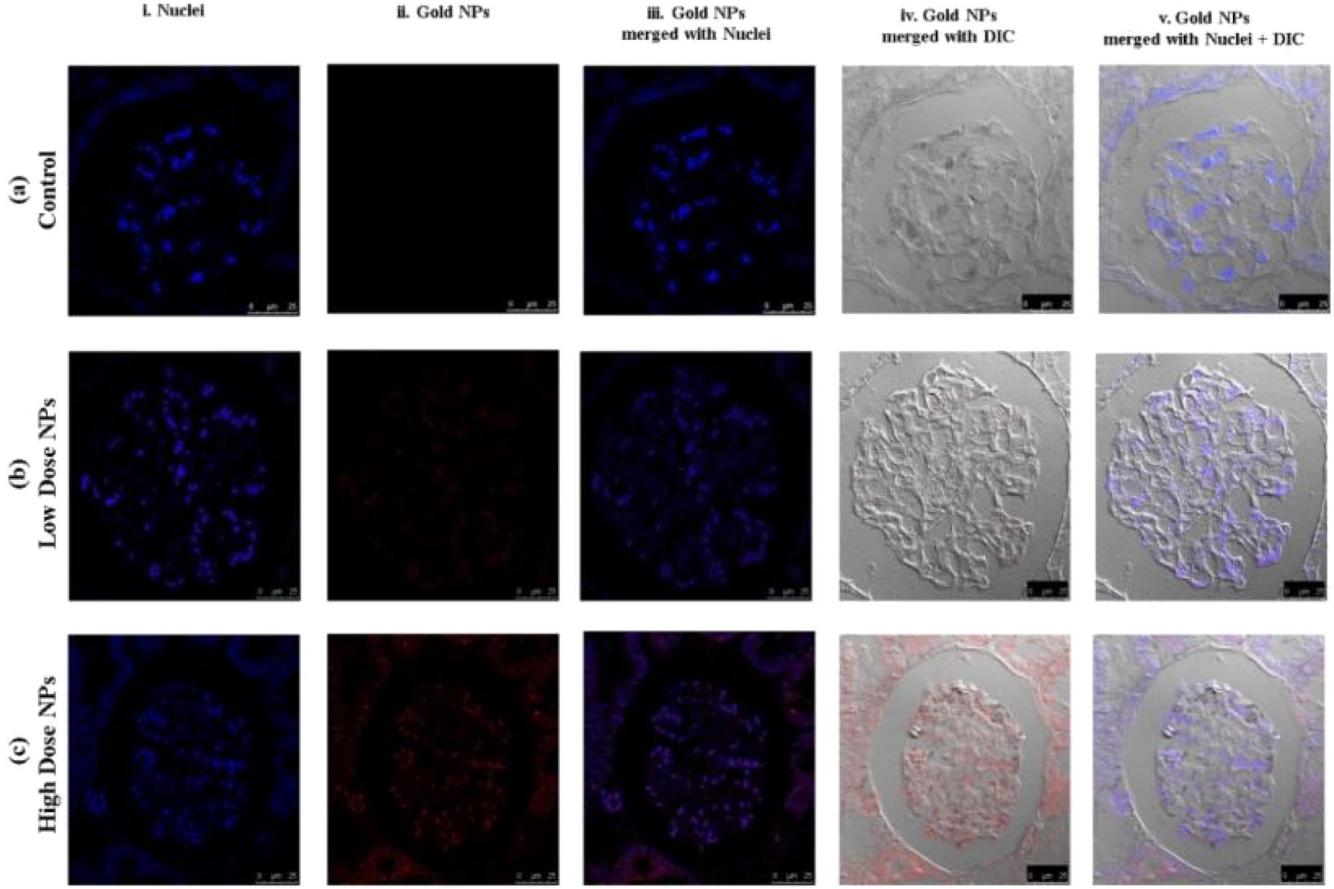
In vivo biodistribution and localization of Au-NPs at different doses (High & Low) in rat kidney tissues. Confocal fluorescence images of rat kidney tissues (**A**) Control group, (**B**) Rat group treated with high dose Au-NPs, and (**C**) Rat group treated with low dose Au-NPs . (i) Blue: Hoechst 33342-stained nuclei. (ii) Red: Gold NPs. (iii) Merged Image from Gold NPs and Nuclei. (iv) DIC mode merged with Gold NPs (v) DIC mode merged with Gold NPs and nuclei. λ_max_ = 505 nm (Hoechst 33342), 555 nm (red autofluorescence from gold NPs), 473 nm (Differential interference contrast [DIC]). (Confocal Laser Scanning Microscope × 63, Scale bar: 25 μm, Scan Mode = XYZ Unidirectional X, Scan Speed = 400 Hz, Pinhole Diameter = 137.1 μm).

**Figure 8:**
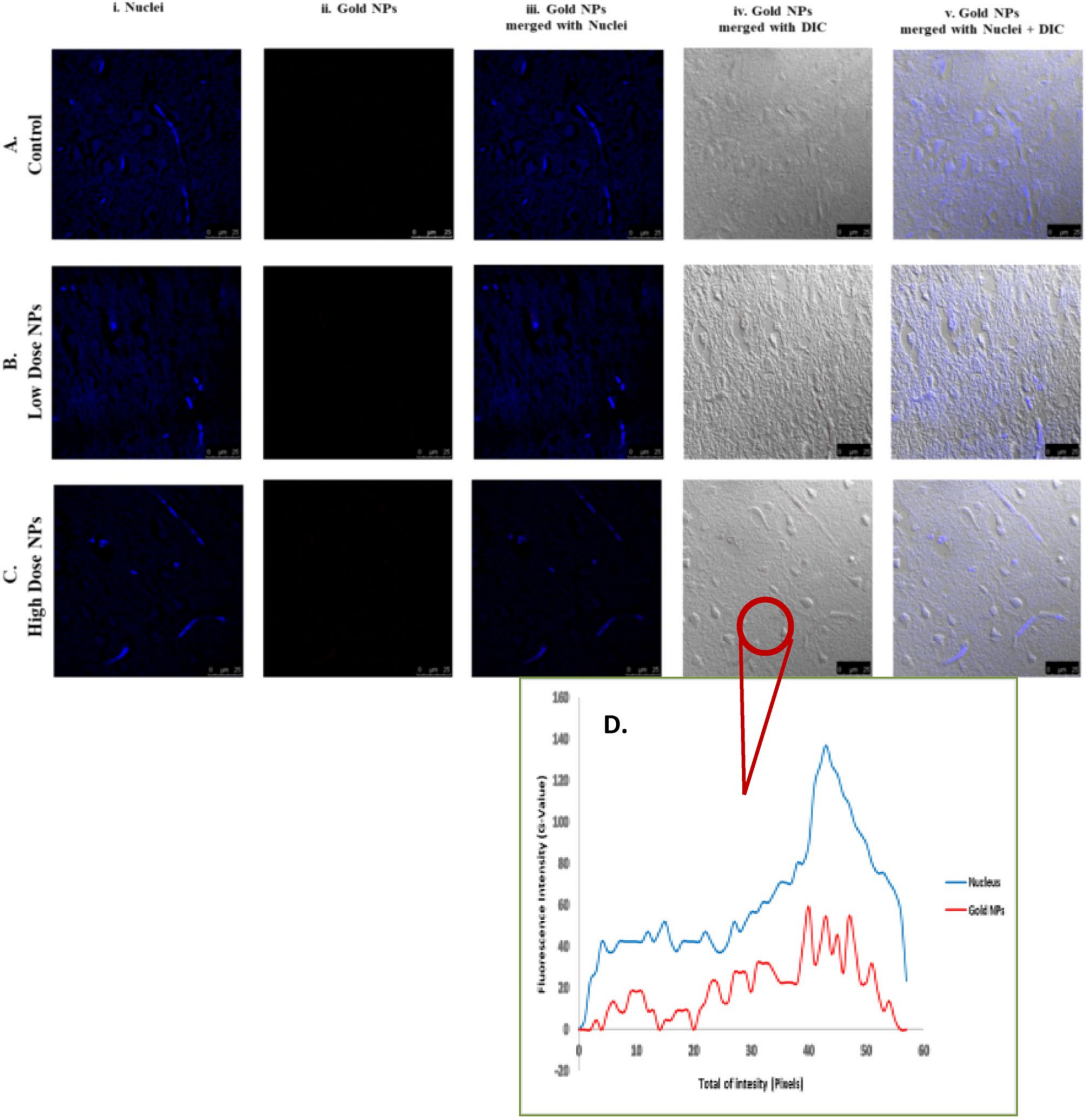
In vivo biodistribution and localization of Au-NPs at different doses (High & Low) in rat brain tissues after intravenous injection. [**A**] Confocal fluorescence images of rat brain tissues (**A**) Control group, (**B**) Rat group treated with high dose Au-NPs, and (**C**) Rat group treated with low Au-NPs . (i) Blue: Hoechst 33342-stained nuclei. (ii) Red: Gold NPs. (iii) Merged Image from Gold NPs and Nuclei. (iv) DIC mode merged with Gold NPs (v) DIC mode merged with Gold NPs and nuclei. λ_max_ = 505 nm (Hoechst 33342), 555 nm (red autofluorescence from gold NPs), 473 nm (Differential interference contrast [DIC]). (Confocal Laser Scanning Microscope × 63, Scale bar: 25 μm, Scan Mode = XYZ Unidirectional X, Scan Speed = 400 Hz, Pinhole Diameter = 137.1 μm). (**D**) The relative intensity profiles revealed the Au-NPs signal completely overlapping with that of the nuclear staining of microglia, showed that although trace amounts of Au-NPs were crossed through the blood brain barrier, they were uptaken by microglia (circled area).

**Figure 9:**
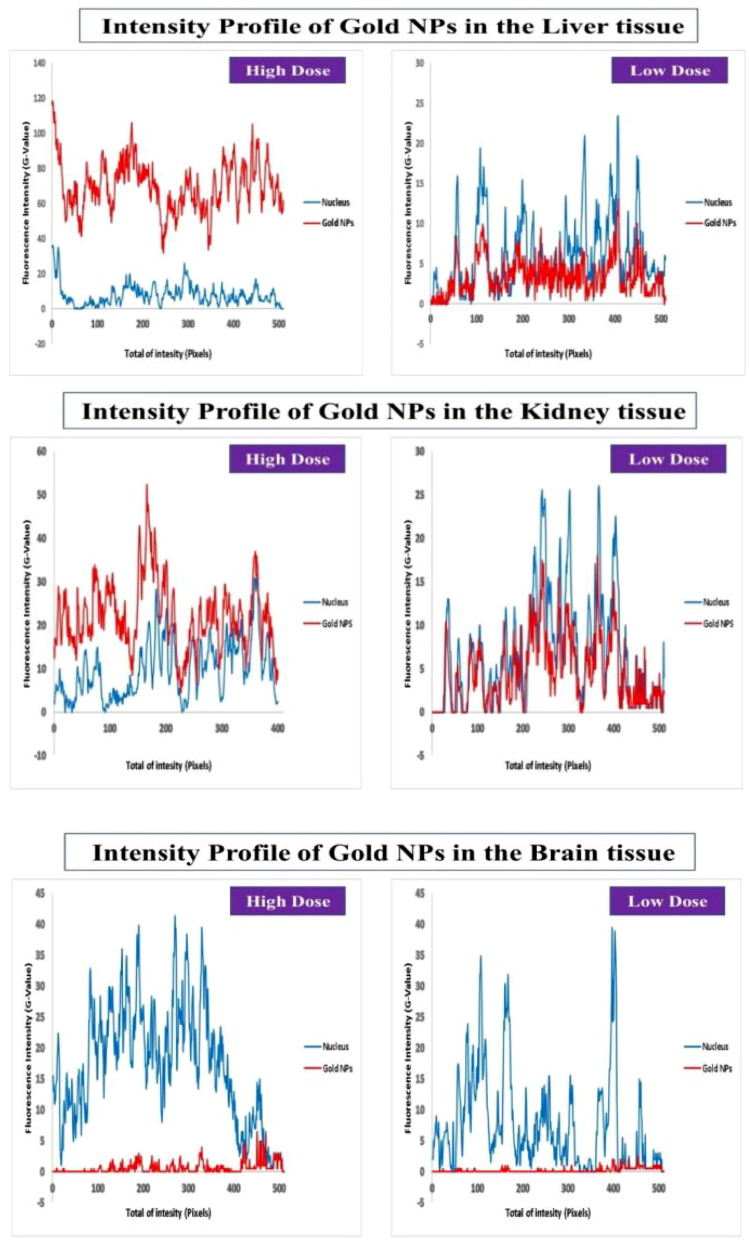
Graph representing the fluorescence intensity profiles of Au-NPs in rat liver, kidney and brain tissues as a function of distance. In liver and kidney tissue, control group showed no red fluorescence because of the absence of Au-NPs, thus acting as a zero-fluorescence base line to exclude the autofluorescence background. The biodistribution of Au-NPs at high dose was significantly higher than that of rat group treated with low dose Au-NPs as evaluated by MFI and mean area % (*p* = *0.0013, p* < *0.0001* respectively). In brain tissue, control group showed no red fluorescence because of the absence of Au-NPs, thus acting as a zero-fluorescence base line to exclude the autofluorescence background. The biodistribution of Au-NPs at high dose was more prominent than that of rat group treated with low dose Gold NPs as evaluated by MFI (*p* = *0.0062*), on the other hand, there was no significant differences between the two treated groups with respect to the mean area % (*p* = *0.3444*). The square represented the high density of Au-NPs in glia cells.

**Figure 10:**
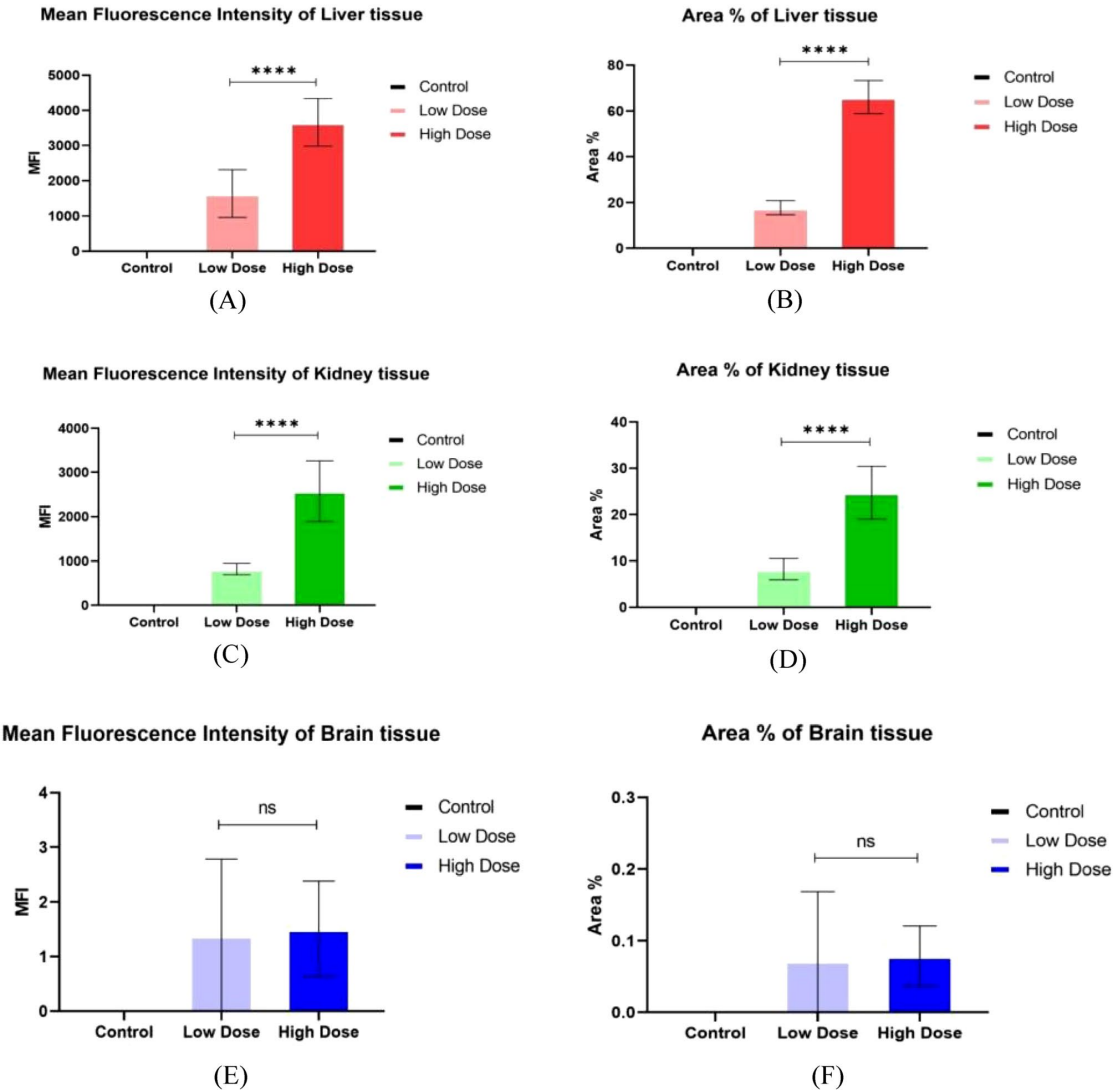
Mean fluorescence intensity (MFI) (**A**) and Mean area % of Au-NPs in rat liver (**B**), Kidney (**C**, **D**) and brain tissues (**E**, **F**) were calculated by using Image J. Data are expressed as mean ± SE; Significance: N.S.: no significance *p ≤ 0.05, **p ≤ 0.01, ***p ≤ 0.001, ****p ≤ 0.001.

Over all, the results revealed that the high dosage of Au-NPs had a relatively greater cellular localization in the liver, kidney, and brain than the low dose (Figs. 6, 7, 8). This was confirmed by the finding that the mean fluorescence intensity (MFI) of Au-NPs in the liver, kidney, and brain tissues that were treated with high dosage Au-NPs (Group 3) were approximately 1.96, 5.75, and 0.9 times greater than that of low dose Au-NPs (Group 2), respectively (Fig. 9). Consequently, the mean localization area (%) was raised by around 4.41, 2.96, and 0.3 times in the liver, kidney, and brain tissues treated with high dosage Au-NPs compared to those treated with low dose Au-NPs, respectively (Fig. 10).

For instance, both the liver sectors and renal tissues, exhibited NPs localization inside the cells of low dose Au-NPs (Group 2). The localization of NPs was more prominent in high dose Au-NPs sectors (Figs. 6, 7). More broadly, these results were supported by fluoresce imaging as shown by arrows in Fig. 6, and also illustrated by the higher MFI and mean area (%) in Fig. 10, reflecting the significant biodistribution of these NPs in all types of liver and renal tissues that received high-dose Au-NPs.

**Figure 11:**
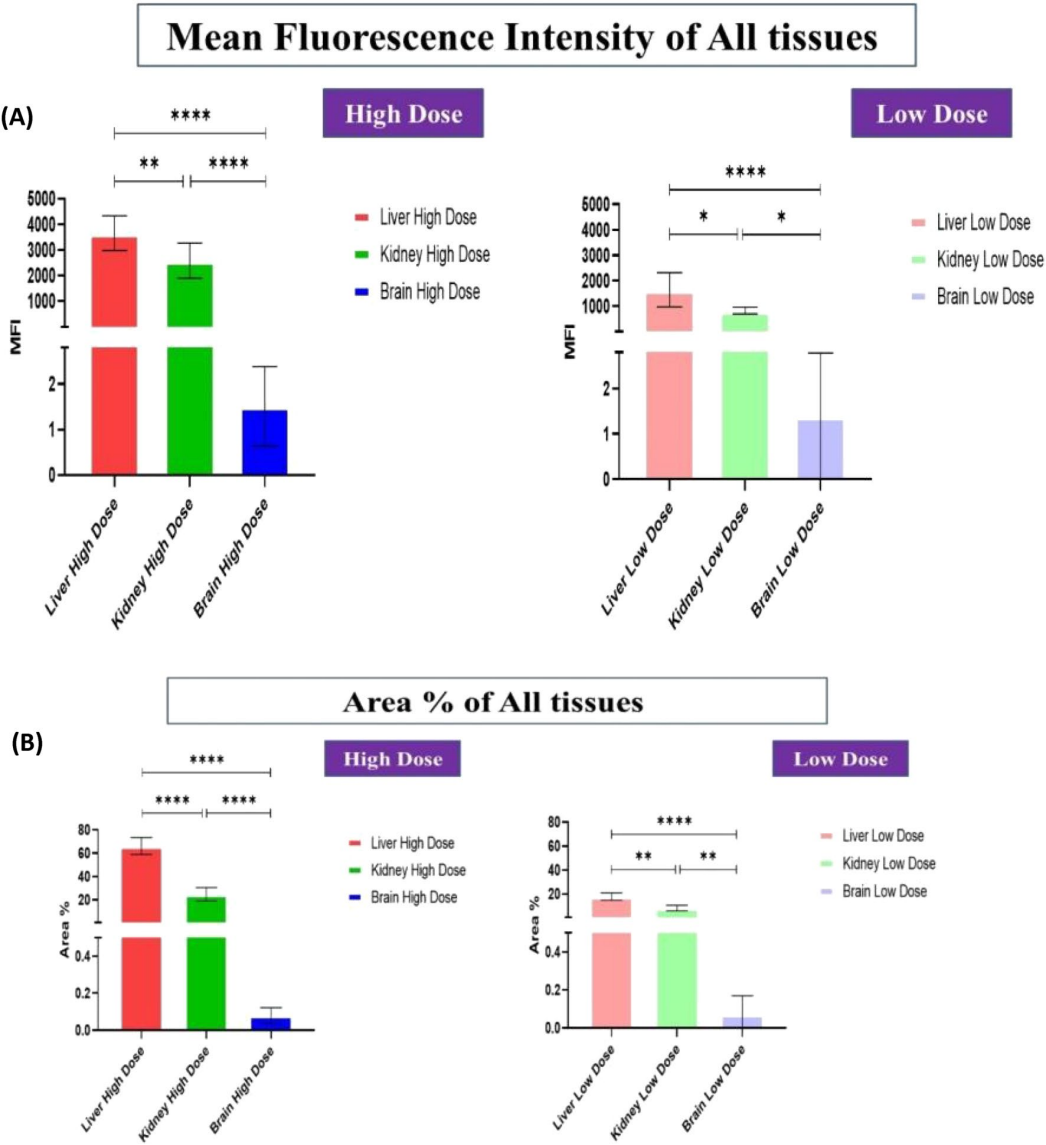
Comparison between the MFI (**A**) and the mean area % (**B**) of the studied tissues. Data were expressed as mean ± SE; Significance: N.S.: no significance *p ≤ 0.05, **p ≤ 0.01, ***p ≤ 0.001, ****p ≤ 0.001.

On the other hand, the brain tissue exhibited the lowest quantity of Au-NPs accumulation compared to other examined tissues due to the fact that the blood brain barrier is a highly selective semipermeable border of endothelial cells that significantly restricts the entry of most molecules at the level of cerebral cortex (Fig. 8). Subsequently, the MFI and mean area (%) confirmed no significant variation between high and low treated Au-NPs doses in brain tissues (Fig. 10). Surprisingly, limited Au-NPs accumulation was seen in the glial cells of the brain of high-dose treated group (Group 3) which was revealed in Fig. 8. In agreement with the previous finding tissue intensity profile (pixel) also proved this (Fig. 11).

## Discussion

Gold nanoparticles (Au-NPs) are among many precious nano-metals with unique surface plasmon resonance (SPR) which attributed their use in near-infrared (NIR) such as the magnetic resonance imaging (MRI), fluorescence imaging, photoacoustic imaging (PAI) and X-ray scatter imaging^32^. Au-NPs have non-immunogenic and low toxicity properties. Au-NPs size is the most important key parameter that influences their systemic toxicity and biological activities^33^. Hence the present study aimed to synthesize ultra-small gold nanoparticles and assess their biological activities in vitro and track their in vivo biodistribution and acute toxicity.

Egyptian propolis extract was chosen to greenly synthesis the ultra-small Au-NPs. Data revealed that the synthesized nanoparticles appeared at 530–538 nm in the UV-scan with 7.8 nm average diameter, 0.385 PDI and − 34.7 mV Z-potential indicating their ultra-small size with great stability and narrow size distribution. FTIR analysis proved the propolis anticipation as reducing, and stabilizing agent with various terminal carboxyl groups. Our results were in accordance with Botteon et al.^20^ who synthesized Au-NPs using Brazilian red propolis with average size 8–15 nm with narrow particle size distribution. This proves the superior activity of the prepared Au-NPs over the previously reported one in accordance to the smaller diameter size and higher stability. In another avenue, as compared to neutral or positively charged particles, the higher negative potential may increase the nanoparticles circulation time in vivo^34^ which was considered as an added value to the synthesized Au-NPs in the present work. Hence appeared the importance of assessing the Au-NPs stability through determining the zeta potential changes in different biological fluids. By increasing the zeta potential (either positive or negative) then the particles have the sufficient repulsive energy to increase the particles homogeneity, dispersity and stability. Our results revealed the good stability of the prepared Au-NPs in different biological fluids (around ± 50 mV) as described by Reznickova et al.^26^.

The antimicrobial activity of the synthesized ultra-small Au-NPs was evaluated through disc diffusion, MIC and microbial lethality curve methods. Data revealed that the prepared Au-NPs had a potent antimicrobial activity with MIC value 4 µg/mL and 45 mm inhibition zone diameter against *Staphylococcus epidermidis*. Microbial lethality curve proved the potent activity of the synthesized nanoparticles with complete eradication of the microbial growth after 4, 12 and 20 h against MRSA, *K. pneumoniae* and *C. albicans* respectively. On the other hand, the inhibitory effect of the ultra-small Au-NPs against *M. tuberculosis* clinical strains varied between 90 and 99% while the ultra-small gold nanoparticles effectively inhibited the growth of the standard strain *M. tuberculosis* (ATCC 25177) with 98.4%. The antibacterial mode of action of Au-NPs was discussed by Mohamed et al.^35^ who reported that gold nanoparticles have a bactericidal effect through bacterial cell wall penetration, aggregation and vacuoles formation inside the cytoplasm. In line with the current findings, Zawrah et al.^36^ reported that fluconazole-coated Au-NPs have shown improved antifungal efficacy against *Aspergillus niger*, *Candida albicans*, and *Aspergillus flavus*^36^. Avan et al.^37^ revealed that Au-NPs with 13 nm diameter have a broad range of antimicrobial activity against multiple microorganisms at various doses. Previous researches have indicated that plant-mediated metal nanoparticles play an important role in medication administration and may be utilized to combat a wide range of bacteria due to their effective antimicrobial characteristics^38–40^. Abdel-Aziz et al.^38^ studied the in vitro anti-mycobacterial activity of Au-NPs (11–17.5 nm diameters) and stated that the MIC value reached 1.95 µg/mL. However, Govindaraju et al.^39^ revealed that the sensitive strain of *M. tuberculosis* showed lower MIC value upon treatment with Au-NPs reached 10 μg/mL compared to rifampicin resistant strains (zero inhibition). On the contrary, Singh et al.^40^ reported that Au-NPs had no anti-mycobacterium activity at different concentrations up to 100 µg/mL.

Au-NPs of various shapes and sizes with desirable characteristics were produced using chemical synthesis technologies, allowing for multimodal cancer treatment with extended anti-tumor action^41^. Huang et al.^42^ demonstrated that the ultra-small Au-NPs had marvelous advantages in tumor penetration in vivo. In recent study, Brazilian red propolis was used for synthesis of Au-NPs which exhibited potent anticancer activity with IC50 (> 43 µg/mL) against T24 and PC3 cells^20^. Other recent studies declared that IC50 values of green synthesized Au-NPs against liver and breast cancer cell lines (HepG-2 and MCF-7, respectively) were 23.0–59.62 µg/mL and 50–98 µg/mL, respectively^43–45^. Meanwhile, in the present study, the synthesized ultra-small Au-NPs showed remarkable cytotoxic effect against Caco-2, HepG-2 and MDA-MB 231 cells (IC50 ≤ 23.8 µg/mL). Different studies attributed the Au-NPs cellular uptake to the particle size where the ultra-small nanoparticles size means higher cellular uptake, higher probability of cancer cells nucleus penetration and lower toxicity than larger Au-NPs^42,46^. The anticancer mechanism of Au-NPs is mainly through overgeneration of ROS which mediates apoptosis via loss mitochondrial potential, resulting in up-regulation of proapoptotic genes (e.g., BAX) and activation of caspase cascade with suppression of Bcl2. Also, ROS inhibits AKT which up-regulates the expression of p53 and its downstream genes (e.g., BAX and p21)^47–49^. Our recent study is consistent with the above-mentioned findings, Au-NPs significantly elevated ROS level in all investigated cancer cell lines and subsequently, these as-prepared NPs lowered the expression of Bcl2 and enhanced the expression of p53 and BAX by 3–6 folds (Fig. 4).

To achieve effective therapeutic capabilities, biomedical applications demand the use of less toxic Au-based nanoparticles. Many investigations were carried out to determine the toxicity level and thus the safety of these formulations for human use. Clearly, the physicochemical characteristics of these particles, their biodistribution and cellular absorption, as well as the cell line or animal model, directly influence the harmful consequences^1^.

In vivo biodistribution and toxicity of ultra-small Au-NPs were demonstrated in our experimental rats using histopathology and confocal fluorescence microscopy imaging. Animals' healthy behavior, as well as the absence of acute toxicity on the liver, kidney, and brain, proved the Au-NPs' low dose (10 mg/kg) safety as previously recommended by Bailly et al.^8^*.* Moreover, Pannerselvam et al.^50^ confirmed that histopathology and density of Au-NPs modulation in tissue biodistribution was the most important investigation for in vivo toxicity. Liu et al.^51^ findings offered a scientific foundation for researchers to choose the best animal model and dosage paradigms for future nanomaterial investigations, therefore increasing the research's applicability to humans. Generally, intraperitoneal injection of Au-NPs was reported to be less hazardous than oral delivery^4^.

Zheng et al.^52^ proved that the clearance of Au-NPs with average size 6 nm was by blood filtration through the kidney to the bladder. Loynachan et al.^53^ declared that the ultra-small Au-NPs could be completely cleared through the liver and kidney which may explain the relatively high Au-NPs biodistribution in the aforementioned tissues. Ibrahim et al.^2^ reported no toxic effect on the kidneys of Swiss albino mice 24 h after intraperitoneal injection of 50 nm hexagonal-shaped Au-NPs at a dose of 170 mg/kg bodyweight, which in consistent with the observed low toxicity towards kidney cells for the synthesized ultra-small gold Nanospheres. Consequently, it is clear that size, type of coating material and dose of Au-NPs affect their accumulation and toxicity in renal tissues^54^.

The present study exhibited few or more changes in comparison to normal in low-dose Au-NPs injections either in the liver or kidney tissues. Further investigations revealed that high dose Au-NPs produced more pathological changes which are consistent with the findings of Ibrahim et al.^2^. Indeed, subsequent to the NPs treatment, the liver has been generally identified as a critical target organ for nanoparticle accumulation that occurs primarily inside Kupffer cells and internalized by endocytosis pathways^5^.

According to Kumar et al.^46^, who used confocal fluorescence microscopic studies to confirm the same histopathological observations of Au-NPs in experimental models, the cellular internalization and localization of Au-NPs was emphasized. The previously mentioned characteristics were used to validate their interactions with cells using confocal fluorescence microscopy, which revealed a buildup of significant Au-NPs in high doses that was more evident in kupper cells and represented histopathological damage. Ganeshkumar et al.^54^ demonstrated that in vivo biodistribution of Au-NPs after intraperitoneal injection (3380 μg Au-NPs) in male Wistar rats, where the major accumulation of these nanoparticles was noticed in liver followed by kidneys and lungs. However, the fact that the nanoparticles only changed some metabolic activities as measured by the MTT cytotoxicity assay, without affecting mitochondrial membrane potentiality, lysosomal integrity, ROS/RNS production, energetic and redox status, or activating apoptosis, suggested that Au-NPs could only cause early-phase cytotoxicity without disrupting the cells' normal functioning^10^.

Unlike blood capillaries elsewhere in the body, BBB is considered as major challenge, formed by the endothelial cells that line cerebral microvessels. The structure of the BBB is characterized by the tight-junctions that are highly resistant to the exchange of substances between the blood and the nervous tissue^13^. Supporting to pervious findings; surface modifications of Au-NPs were required to add targeting moieties such as ligands, antibodies, and other directing agents to improve their transport across the blood brain barrier and selectively targeting the brain tissues^11,12^. This reflects the NPs’ lack of toxicity in nerve tissue, particularly the cerebral cortex^13^. According to our histopathogical findings, there was no apparent impairment or inflammation in the cerebral cortex of the brain. Confocal imaging indicated that at high doses of Au-NPs, non-significant bioaccumulation of Au-NPs was detected in glial cells rather than brain tissue. Au-NPs have unique charge and surface characteristics offered by its characteristic surface Plasmon spectral pattern, which can be a key factor in this limited accumulation in glial cells as mentioned by Barthakur^55^.

Finally, our results were in line with the pharmacokinetic profile described by Bailly et al.^8^. Since the doses used in animal studies are generally higher than the actual doses humans are exposed to, and since nanoparticle pharmacokinetics is dose-dependent, as mentioned by Cheng et al.^3^ study, thus the low dose (10 mg/kg) of the greenly prepared ultra-small Au-NPs is considered safe for biomedical application.

## Conclusion

Egyptian propolis extract can be used as efficient reducing agent for ultra-small Au-NPs synthesis. Ultra-small Au-NPs were examined using UV-scan, TEM, SEM, EDX, FTIR and DLS modalities. It was demonstrated that the prepared nanoparticles had an average size less than 10 nm with great purity, stability and monodispersity. Ultra-small Au-NPs showed potent biological activities (antimicrobial, anti-mycobacterium and anticancer). The acute toxicity profile of the prepared nanoparticles proved that the Au-NPs low dose (10 mg/kg) can be used safely in several biomedical applications. The in vivo biodistribution of the ultra-small gold nanoparticles also proved the biosafety of the Au-NPs low dose.

## Supplementary Information

Supplementary Figures.

## References

[1] Lopez-Chaves C, Soto-Alvaredo J, Montes-Bayon M, Bettmer J, Llopis J, Sanchez-Gonzalez C (2018). Gold nanoparticles: Distribution, bioaccumulation and toxicity. In vitro and in vivo studies. Nanomed. Nanotechnol. Biol. Med..

[2] Ibrahim KE, Al-Mutary MG, Bakhiet AO, Khan HA (2018). Histopathology of the liver, kidney, and spleen of mice exposed to gold nanoparticles. Molecules.

[3] Cheng YH, Riviere JE, Monteiro-Riviere NA, Lin Z (2018). Probabilistic risk assessment of gold nanoparticles after intravenous administration by integrating in vitro and in vivo toxicity with physiologically based pharmacokinetic modeling. Nanotoxicology.

[4] Wu T, Tang M (2018). Review of the effects of manufactured nanoparticles on mammalian target organs. J. Appl. Toxicol..

[5] Haute DV, Berlin JM (2017). Challenges in realizing selectivity for nanoparticle biodistribution and clearance: Lessons from gold nanoparticles. Ther. Deliv..

[6] Miller MA (2001). Gender-based differences in the toxicity of pharmaceuticals—The Foodand Drug Administration's perspective. Int. J. Toxicol..

[7] Cassano D, Summa M, Pocoví-Martínez S, Mapanao AK, Catelani T, Bertorelli R, Voliani V (2019). Biodegradable ultrasmall-in-nano gold architectures: Mid-period in vivo distribution and excretion assessment. Part. Part. Syst. Charact..

[8] Bailly AL, Correard F, Popov A, Tselikov G, Chaspoul F, Appay R (2019). In vivo evaluation of safety, biodistribution and pharmacokinetics of laser-synthesized gold nanoparticles. Sci. Rep..

[9] Bin-Jumah MN, Al-Abdan M, Al-Basher G, Alarifi S (2020). Molecular mechanism of cytotoxicity, genotoxicity, and anticancer potential of green gold nanoparticles on human liver normal and cancerous cells. Dose-Response.

[10] Enea M, Pereira E, Peixoto de Almeida M, Araújo AM, Bastos MDL, Carmo H (2020). Gold nanoparticles induce oxidative stress and apoptosis in human kidney cells. Nanomaterials.

[11] Morshed RA, Muroski ME, Dai Q, Wegscheid ML, Auffinger B, Yu D, Lesniak MS (2016). Cell-penetrating peptide-modified gold nanoparticles for the delivery of doxorubicin to brain metastatic breast cancer. Mol. Pharm..

[12] Raliya R, Saha D, Chadha TS, Raman B, Biswas P (2017). Non-invasive aerosol delivery and transport of gold nanoparticles to the brain. Sci. Rep..

[13] Khongkow M, Yata T, Boonrungsiman S, Ruktanonchai UR, Graham D, Namdee K (2019). Surface modification of gold nanoparticles with neuron-targeted exosome for enhanced blood–brain barrier penetration. Sci. Rep..

[14] Barthakur M, Kalita P, Mondal S (2020). Modulation of astrocytic membrane potential using citrate stabilized gold nanoparticle to control brain hyper-excitability. AIP Conf. Proc..

[15] Talarska P, Boruczkowski M, Żurawski J (2021). Current knowledge of silver and gold nanoparticles in laboratory research—Application, toxicity. Cell. Uptake. Nanomater..

[16] Hamed MT, Bakr BA, Shahin YH, Elwakil BH, Abu-Serie MM, Aljohani FS, Bekhit AA (2021). Novel synthesis of titanium oxide nanoparticles: Biological activity and acute toxicity study. Bioinorg. Chem. Appl..

[17] López BGC, Schmidt EM, Eberlin MN, Sawaya AC (2014). Phytochemical markers of different types of red propolis. Food Chem..

[18] Roy N, Mondal S, Laskar RA, Basu S, Mandal D, Begum NA (2010). Biogenic synthesis of Au and Ag nanoparticles by Indian propolis and its constituents. Colloids Surf. B.

[19] Gatea F, Teodor ED, Seciu AM, Covaci OI, Mănoiu S, Lazăr V, Radu GL (2015). Antitumour, antimicrobial and catalytic activity of gold nanoparticles synthesized by different pH propolis extracts. J. Nanopart. Res..

[20] Botteon CEA, Silva LB, Ccana-Ccapatinta GV, Silva TS, Ambrosio SR, Veneziani RCS, Marcato PD (2021). Biosynthesis and characterization of gold nanoparticles using Brazilian red propolis and evaluation of its antimicrobial and anticancer activities. Sci. Rep..

[21] Elnaggar YS, Elwakil BH, Elshewemi SS, El-Naggar MY, Bekhit AA, Olama ZA (2020). Novel Siwa propolis and colistin-integrated chitosan nanoparticles: Elaboration; in vitro and in vivo appraisal. Nanomedicine.

[22] Elwakil B, Shaaban MM, Bekhit AA, El-Naggar MY, Olama ZA (2021). Potential anti-COVID-19 activity of Egyptian propolis using computational modeling. Futur. Virol..

[23] Dobrucka R (2017). Synthesis of titanium dioxide nanoparticles using Echinacea purpurea herba. Iran. J. Pharm. Res. IJPR.

[24] Yasmin A, Ramesh K, Rajeshkumar S (2014). Optimization and stabilization of gold nanoparticles by using herbal plant extract with microwave heating. Nano Convergence.

[25] Zheng Y, Zhang J, Zhang R, Luo Z, Wang C, Shi S (2019). Gold nano particles synthesized from Magnolia officinalis and anticancer activity in A549 lung cancer cells. Artif. Cells Nanomed. Biotechnol..

[26] Reznickova A, Slavikova N, Kolska Z, Kolarova K, Belinova T, Kalbacova MH, Cieslar M, Svorcik V (2019). PEGylated gold nanoparticles: Stability, cytotoxicity and antibacterial activity. Colloids Surf. A.

[27] European Centre for Disease Prevention and Control (2016). Handbook on TB Laboratory Diagnostic Methods for the European Union.

[28] Mosmann T (1983). Rapid colorimetric assay for cellular growth and survival: Application to proliferation and cytotoxicity assays”. J. Immunol. Methods.

[29] Younes NRB, Amara S, Mrad I, Ben-Slama I, Jeljeli M, Omri K, Sakly M (2015). Subacute toxicity of titanium dioxide (TiO_2_) nanoparticles in male rats: Emotional behavior and pathophysiological examination. Environ. Sci. Pollut. Res..

[30] Ahmed SM, Mohammed MZ, Mahmoud AA (2018). The role of gold nanoparticles on taxol-induced renal cortex damage in adult male albino rats. Egypt. J. Histol..

[31] Balasubramanian SK, Jittiwat J, Manikandan J, Ong CN, Liya EY, Ong WY (2010). Biodistribution of gold nanoparticles and gene expression changes in the liver and spleen after intravenous administration in rats. Biomaterials.

[32] Mukherjee S, Sau S, Madhuri D, Bollu VS, Madhusudana K, Sreedhar B (2016). Green synthesis and characterization of monodispersed gold nanoparticles: Toxicity study, delivery of doxorubicin and its bio-distribution in mouse model. J. Biomed. Nanotechnol..

[33] Fan M, Han Y, Gao S, Yan H, Cao L, Li Z, Zhang J (2020). Ultrasmall gold nanoparticles in cancer diagnosis and therapy. Theranostics.

[34] Kotcherlakota R, Nimushakavi S, Roy A, Yadavalli HC, Mukherjee S, Haque S, Patra CR (2019). Biosynthesized gold nanoparticles: In vivo study of near-infrared fluorescence (NIR)-based bio-imaging and cell labeling applications. ACS Biomater. Sci. Eng..

[35] Mohamed MM, Fouad SA, Elshoky HA, Mohammed GM, Salaheldin TA (2017). Antibacterial effect of gold nanoparticles against *Corynebacterium pseudotuberculosis*. Int. J. Veterinary Sci. Med..

[36] Zawrah MF, El-Moez SA, Center D (2011). Antimicrobial activities of gold nanoparticles against major foodborne pathogens. Life Sci. J..

[37] Avan, E. D., Quadry, R. O., Ikenna-Ossai, C. N. & Okolie, N. P. Effects of Annona muricata biofunctionalized gold nanoparticles on erythrocyte osmotic fragility and hematological profile in rat model. *Covenant J. Phys. Life Sci. (Special Edition)*. **1**(2), 33–45 (2018).‏

[38] Abdel-Aziz MM, Elella MHA, Mohamed RR (2020). Green synthesis of quaternized chitosan/silver nanocomposites for targeting mycobacterium tuberculosis and lung carcinoma cells (A-549). Int. J. Biol. Macromol..

[39] Govindaraju K, Vasantharaja R, Suganya KU, Anbarasu S, Revathy K, Pugazhendhi A, Singaravelu G (2020). Unveiling the anticancer and antimycobacterial potentials of bioengineered gold nanoparticles. Process Biochem..

[40] Singh R, Nawale LU, Arkile M, Shedbalkar UU, Wadhwani SA, Sarkar D, Chopade BA (2015). Chemical and biological metal nanoparticles as antimycobacterial agents: A comparative study. Int. J. Antimicrob. Agents.

[41] Hammami I, Alabdallah N (2021). Gold nanoparticles: Synthesis properties and applications. J. King Saud Univ.-Sci..

[42] Huang K, Ma H, Liu J, Huo S, Kumar A, Wei T, Liang XJ (2012). Size-dependent localization and penetration of ultrasmall gold nanoparticles in cancer cells, multicellular spheroids, and tumors in vivo. ACS Nano.

[43] Lia L, Zhangb W, Devanatha V, Seshadric D, Guangshao C (2019). Synthesis and characterization of gold nanoparticles from *Marsdenia tenacissima* and its anticancer activity of liver cancer HepG2 cells. Nanomed. Biotechnol..

[44] Al-Radadi NS (2021). Facile one-step green synthesis of gold nanoparticles (AuNp) using licorice root extract: Antimicrobial and anticancer study against HepG2 cell line. Arab. J. Chem..

[45] Vijayakumar S (2019). Eco-friendly synthesis of gold nanoparticles using fruit extracts and in vitro anticancer studies. J. Saudi Chem. Soc..

[46] Kumar A, Ma H, Zhang X, Huang K, Jin S, Liu J, Liang XJ (2012). Gold nanoparticles functionalized with therapeutic and targeted peptides for cancer treatment. Biomaterials.

[47] Li P, Nijhawan D, Budihardjo I, Srinivasula SM, Ahmad M, Alnemri ES, Wang X (1997). Cytochrome c and dATP-dependent formation of Apaf-1/caspase-9 complex initiates an apoptotic protease cascade. Cell.

[48] Akter M, Sikder MT, Rahman MM, Ullah AA, Hossain KFB, Banik S (2018). A systematic review on silver nanoparticles-induced cytotoxicity: Physicochemical properties and perspectives. J. Adv. Res..

[49] Mukherjee S, Dasari M, Priyamvada S, Kotcherlakota R, Bollu VS, Patra CR (2015). A green chemistry approach for the synthesis of gold nanoconjugates that induce the inhibition of cancer cell proliferation through induction of oxidative stress and their in vivo toxicity study. J. Mater. Chem. B.

[50] Pannerselvam B, Devanathadesikan V, Alagumuthu TS, Kanth SV, Thangavelu KP (2020). Assessment of in-vivo biocompatibility evaluation of phytogenic gold nanoparticles on Wistar albino male rats. IET Nanobiotechnol..

[51] Liu YC, Lin MTY, Ng AHC, Wong TT, Mehta JS (2020). Nanotechnology for the treatment of allergic conjunctival diseases. Pharmaceuticals.

[52] Zhou C, Long M, Qin Y, Sun X, Zheng J (2011). Luminescent gold nanoparticles with efficient renal clearance. Angew. Chem. Int. Ed..

[53] Loynachan CN, Soleimany AP, Dudani JS, Lin Y, Najer A, Bekdemir A, Stevens MM (2019). Renal clearable catalytic gold nanoclusters for in vivo disease monitoring. Nat. Nanotechnol..

[54] Ganeshkumar M, Ponrasu T, Raja MD, Subamekala MK, Suguna L (2014). Green synthesis of pullulan stabilized gold nanoparticles for cancer targeted drug delivery. Spectrochim. Acta Part A Mol. Biomol. Spectrosc..

[55] Barthakur M (2021). Nanoparticles for modulation of astrocyte physiology: A possible therapeutic agent to control brain hyper-excitability. New Ideas Concerning Sci. Technol..

