# Supplementary figures and images for: In vivo bio-distribution and acute toxicity evaluation of greenly synthesized ultra-small gold nanoparticles with different biological activities
Source: Sci Rep. 2022 Apr 15;12:6269. doi: 10.1038/s41598-022-10251-7 (PMC9012758; doi:10.1038/s41598-022-10251-7)

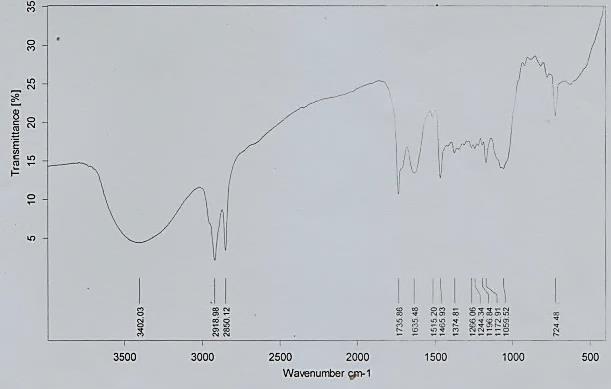


Figure S1. FTIR of the propolis extract


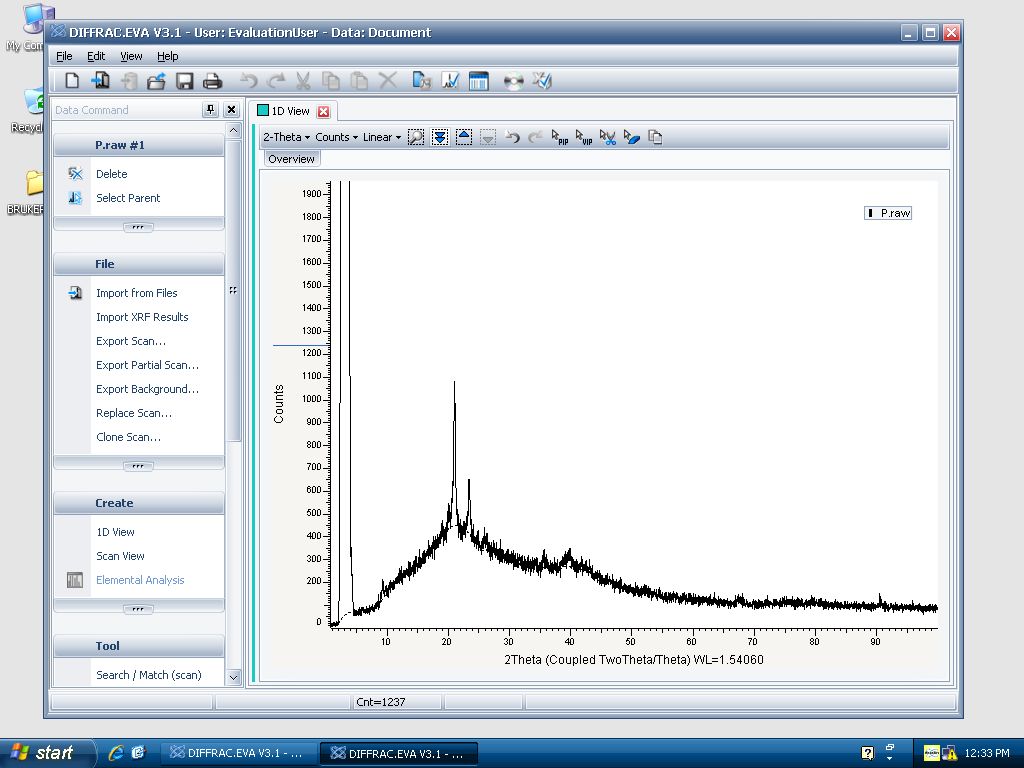


Figure S2. XRD of the propolis extract

Supplement: Supplementary file 1 — Supplementary Figures. [file 41598_2022_10251_MOESM1_ESM.docx]
